# The Sleep–Wake Cycle in the Nicotinic Alpha-9 Acetylcholine Receptor Subunit Knock-Out Mice

**DOI:** 10.3389/fncel.2017.00302

**Published:** 2017-10-10

**Authors:** Natalia Madrid-López, Jorge Estrada, Javier Díaz, Alejandro Bassi, Paul H. Délano, Adrián Ocampo-Garcés

**Affiliations:** ^1^Laboratorio de Sueño y Cronobiología, Programa de Fisiología y Biofísica, Instituto de Ciencias Biomédicas, Facultad de Medicina, Universidad de Chile, Santiago, Chile; ^2^Departamento de Neurociencia, Facultad de Medicina, Universidad de Chile, Santiago, Chile; ^3^Departamento de Otorrinolaringología, Hospital Clínico de la Universidad de Chile, Santiago, Chile

**Keywords:** nicotinic alpha 9 acetylcholine receptor, sleep chronobiology, delta EEG buildup, circadian, sleep homeostasis, photic masking

## Abstract

There is a neural matrix controlling the sleep–wake cycle (SWC) embedded within high ranking integrative mechanisms in the central nervous system. Nicotinic alpha-9 acetylcholine receptor subunit (alpha-9 nAChR) participate in physiological processes occurring in sensory, endocrine and immune systems. There is a relationship between the SWC architecture, body homeostasis and sensory afferents so that disruption of afferent signaling is expected to affect the temporal organization of sleep and wake states. The analysis of the SWC of 9 nAChR knock-out animals may help to reveal the contribution of alpha-9 nAChR to sleep chronobiological determinants. Here we explore the polysomnogram in chronically implanted alpha-9 nAChR knock-out (KO) and wild-type (WT) individuals of the hybrid CBA/Sv129 mouse strain. Records were obtained in isolation chambers under a stable 12:12 light:dark cycle (LD). To unmask the 24-h modulation of the SWC a skeleton photoperiod (SP) protocol was performed. Under LD the daily quota (in %) of wakefulness (W), NREM sleep and REM sleep obtained in KO and WT animals were 45, 48 and 7, and 46, 46 and 8 respectively. Both groups exhibit nocturnal phase preference of W as well as diurnal and unimodal phase preference of NREM and REM sleep. The acrophase mean angles of KO vs. WT genotypes were not different (Zeitgeber Time: 6.5 vs. 14.9 for W, 4.3 vs. 2.8 for NREM sleep and 5.3 vs. 3.4 for REM sleep, respectively). Transference to SP do not affect daily state quotas, phase preferences and acrophases among genotypes. Unmasking phenomena of the SWC such as wake increment during the rest phase under SP was evident only among WT mice suggesting the involvement of retinal structures containing alpha-9 nAChR in masking processes. Furthermore, KO animals exhibit longer NREM and REM sleep episodes that is independent of illumination conditions. Consolidated diurnal NREM sleep contributed to obtain higher values of NREM sleep delta-EEG activity among KO mice during rest phase. In conclusion, circadian and sleep homeostatic aspects of the SWC are operative among alpha-9 nAChR KO animals. We propose that alpha-9 nAChR participate in retinal signaling processes responsible of the positive masking of sleep by light.

## Introduction

The temporal organization of the sleep–wake cycle (SWC) of mammals exhibit specie-specific characteristics. Mice and most murids including rats and hamsters exhibit nocturnal phase preference of activity determined by circadian processes, where wakefulness is concentrated during the dark phase and sleep states during the light phase (Vivaldi et al., 1994; Yasenkov and Deboer, 2012). A typical feature of small mammals is the fragmentation of sleep and wake states in relatively short lasting episodes that alternate during the whole 24 h time frame with longer episodes of wakefulness during the active phase (the dark phase for nocturnal mammals) and consolidated episodes of sleep during the rest phase (Le Bon et al., 2007). Mammals exhibit relatively stable amount of sleep states cumulated during the 24-h period, a property of SWC physiology that evidences sleep homeostatic mechanisms that preserve daily sleep and wake quotas (Dijk, 2009). Together with the circadian effect of the light-dark cycle that determines the quotidian phase adjustment enabling the photic entrainment of the circadian system, retinal illumination also exerts direct effect on sleep–wake state generators, in a phenomenon known as photic masking (Hubbard et al., 2013). Nocturnal mammals exhibit increments in wakefulness when subjected to darkness, and, conversely, sleep augments in response to light stimulation. The direct effect of the visual system on sleep state generators has been supposed to rely on non-image forming proyections of a subpopulation of retinal ganglion cells termed intrinsically photosensitive (ipRGCs, Pickard and Sollars, 2011).

Mice strains share main features of the SWC (nocturnal preference of wakefulness, sleep state fragmentation, sleep compensatory rebound after sleep deprivation) whereas other variables such as sleep state quotas, strength of circadian modulation or rate of sleep compensation exhibit significant strain differences (Franken et al., 1999). Researchers took advantage of the variability observed among mice by exploring genetic determinants of circadian and homeostatic mechanisms involved in mammalian sleep regulation. Reports on Quantitative Trait Loci (QTL) analysis have unveiled a complex relationship between genotype and several SWC parameters (Franken et al., 2001), where unexpected interactions between genotype and chronobiological determinants such as phase of day may emerge.

Here we explore the temporal profile of the SWC in mice where the gene of the alpha 9-Nicotinic Acetylcholine Receptor (alpha 9-nAChR) was knocked-out (KO mice) (Vetter et al., 1999). It is known the role of alpha 9-nAChR in the auditory efferent system of mammals where it participates in the top–down modulation of complex cognitive processes (Terreros et al., 2016). The involvement of alpha 9-nAChR in integrative, stress and reward related behavior, and endocrine processes has been recently reported (Mohammadi et al., 2017). The distribution of alpha 9-nAChRs extends to sensory (auditory, vestibular and dorsal root ganglia), immune system, endocrine glands such as hypophysis and adrenal gland (Simmons and Morley, 1998; Lips et al., 2002; Colomer et al., 2010; Mohammadi et al., 2017). Evidences of altered 24-h modulation of activity has been described in KO mice (129/SvEv strain) by the “corner visit” method, suggesting the involvement of alpha 9-nAChR in circadian physiology (Mohammadi et al., 2017). Furthermore, it has been recently demonstrated the presence of apha 9-nAChRs among retinal neurons (Smith et al., 2014), including bipolar, amacrine, and ganglion cells. It could be hypothesized that altered retinal physiology may be related to the proposed circadian altered profile observed among KO mice.

The SWC is sustained by an extended neural network distributed within the central nervous system that integrates circadian processes and sensory inputs in a state dependent manner (Mignot, 2008). We would like to explore the role of alpha 9-nAChR signaling in the temporal organization and architecture of the sleep and wake cycle.

## Materials and Methods

Experiments complied with American Physiological Society policies, experimental procedures were approved by the Institutional Ethics Committee, permit number CBA #0684, Facultad de Medicina, Universidad de Chile. Polysomnographic data were obtained from 10 alpha 9-nAChR KO and 6 WT adult mice, aged between 60 and 80 days and weighing between 22 and 30 g at the start of the behavioral training. Alpha 9-nAChR KO mice on a 129S6/SvEv backcrossed to CBA/CaJ background and WT littermates were generously provided by Dr. Douglas Vetter from the University of Mississippi. Genotypes were confirmed before and after the behavioral training by PCR screening of genomic DNA extracted and purified from the tail. Mice were maintained in individual cages under a 12:12 LD schedule, ambient temperature of 21–24°C, with water and food *ad libitum* (LabDiet 5P00, Prolab RMH3000).

### Polysomnographic Records

Animals were chronically implanted under deep ketamine/xylazine anesthesia. Two parietal epidural electrode screws (Fine Science Tools Inc., North Vancouver, BC, Canada) were symmetrically placed for EEG, and two stainless steel neck muscle electrodes (Medwire Corp., Mt. Vernon, NY, United States) for EMG. The electrode array was protected and permanently affixed to the skull by dental acrylic and anchoring cranial screws.

After at least 1 week of postsurgical recovery, animals were placed in the recording environment consisting in sound attenuated isolation chamber and maintained under a 12:12 LD schedule, at an ambient temperature of 21–24°C, with water and food *ad libitum*.

Illumination during the light phase was set close to 300 lux. Recordings were obtained in freely moving animals connected to the sleep recording system by means of a counterbalanced cable attached to a slip-ring (Airflyte Electronics, Bayonne, NJ, United States).

Under 12:12 LD (LD schedule), light phase started at Zeitgeber time (ZT) 00 and ended at ZT11:59. Skeleton photoperiod (SP), consisted of continuous darkness interrupted by two 20 min light pulses, timed at ZT00 (morning pulse) and ZT11:40 (evening pulse), so that light pulses of SP delimit the former light phase presented under 12:12 LD condition (Ocampo-Garcés et al., 2011).

Recordings started after at least 2 days of adaptation to recording conditions. A computer-based data acquisition system sampled, displayed and stored EEG and EMG. Sampling was performed at 250 Hz after analog filtering and conditioning of signals by means of an amplifier (Grass Model 15LT, Astromed, Inc., West Warwick, RI, United States). A computer-based data acquisition system sampled, displayed, and stored EEG and EMG data. The EEG channel was band-pass analog-filtered (0.5–30 Hz) and amplified by a factor of 2500–5000. The band-pass for EMG was band-pass analog-filtered (30–100 Hz) and amplified by a factor of 5000–10000. Both signals were notch-filtered (50 Hz). After this signal conditioning, recordings were sampled at 250 Hz.

### Supervised Automated Sleep Scoring Procedure

**FIGURE 1:**
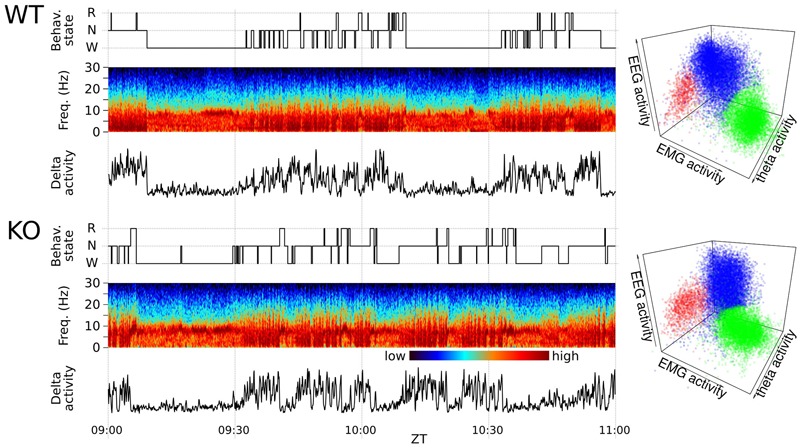
Polysomnographic record and automated sleep scoring of two representative animals. **(Left)** Two-hour fragments from sleep–wake cycles of chronically recorded mice are shown for illustrative purposes. Representative recordings from wild-type (WT) mouse and a Nicotinic Alpha9 Acetylcholine knock-out (KO) as indicated. The panels include from top to bottom: the hypnogram, EEG multitaper spectrogram and delta band activity. **(Right)** Corresponding 5 s-epoch based sleep state 3-D clusters (red = REM, blue = NREM, green = W) of WT and KO animal for one full-day (17280 assignments). Each dot correspond to a 5 s-epoch whose state assignment was automatically determined (see Materials and Methods for further details).

Successive 5-s epochs were assigned to wakefulness (W), NREM sleep or REM sleep by means of an automated sleep scoring system. Automated sleep scoring was performed by analyzing bipolar EEG and EMG channels. Signals were processed to extract their log power spectrum for non-overlapping 5 s epochs. For each epoch, a variable representing EEG activity is calculated as the mean log power in the 1–20 Hz range, a variable representing theta EEG activity is calculated as the maximum of the smoothed log power in the 5.8–8.2 Hz range (0.6 Hz steps, binomial smoothing with 21 coefficients) and a variable representing muscle activity is calculated as the mean log EMG power in the 60–90 Hz range. Each epoch is thus projected in a 3D feature space where three sleep stage related clusters are visible (**Figure 1**). The centroid of each cluster is estimated using an iterative algorithm that maximizes the local density starting from an *a priori* position (high EEG activity and low EMG activity for NREM, high theta activity and low EMG activity for REM, high EMG activity an low EEG activity for W). The distributions of clusters in the neighborhood of the estimated centroid are approximated by Gaussian models assuming ellipsoidal shapes. The covariance matrix of the Gaussian models are used to calculate the Mahalanobis distance from a given epoch to each cluster. Epochs are assigned to a sleep stage according to the minimum Mahalanobis distance to the respective cluster. Epochs that exceed a maximum certainty threshold distance are assigned according to their dynamic context (previous and following stages).

The automated detection procedure was manually corroborated by off-line visual scoring of 3-h recording samples during ZT 4 to ZT 6 (rest phase) and ZT 16 to ZT 18 (active phase) of each experimental day. Visual state scoring procedure assigned each epoch to the state that occupies more than 50% of that epoch. Wakefulness was identified by the presence of desynchronized EEG and phasic EMG activity. NREM sleep consisted of a high-amplitude slow-wave EEG activity accompanied by sleep spindles associated to a low EEG tone. REM sleep was identified by the presence of sustained theta activity coupled with low EEG tone relative to NREM sleep. Epochs scored as NREM or REM sleep with artifacts were excluded from analysis. Cohen’s Kappa coefficient was performed to compare automated and visual scoring procedures (Landis and Koch, 1977). Only experimental days with coefficients superior to 0.6 were considered.

### Experimental Protocol

Mice were uninterruptedly recorded during at least 3 days under a stable LD schedule. After completing LD recording period, animals were transferred to a skeleton photoperiod. Animals were recorded undisturbed during at least 1 week under SP. To avoid transient effects of the transition from LD to SP, selected days under SP were obtained after at least 2 days under SP schedule. For clarity, we denominate the ZT00-ZT11:59 interval as “rest phase,” and the ZT12-ZT23:59 interval as “active phase” for both LD and SP conditions.

### Data Analysis and Statistics

Statistical analysis was carried out using Intercooled Stata 9.2 for Windows (StataCorp, College Station, TX, United States). Circular statistics were performed on custom-made spreadsheets in accordance with procedures described by Zar (1999). The variables analyzed were: amount of wakefulness, NREM, and REM sleep, number of state episodes, and NREM sleep EEG slow-wave activity. Variables may be collapsed into 12-h blocks corresponding to light and dark phases (rest and active phase respectively), 24 consecutive hour bins to analyze and display the state mean curves, and 12 consecutive 2-h blocks to analyze NREM sleep delta activity.

Amounts of wakefulness, NREM, and REM sleep correspond to the number of epochs observed at the corresponding time-window, expressed in minutes. Mean curves were obtained by averaging two (LD or SP) recording days per mouse at the corresponding time resolution. Sleep–wake state acrophases were estimated using mean angle calculation.

For the NREM sleep EEG delta power analysis, only NREM sleep episodes longer than 24 consecutive 5-s epochs (2 min) were considered. EEG analyses were performed in Igor Pro V6.3. Multitaper spectrograms were constructed from EEG signals following the general indications from Prerau (Prerau et al., 2017) and using the “dpss” function from the “multitaper” R package. EEG was divided in 5 s epochs with 2.5 s overlapping. We used the parameters time-bandwidth = 10 and number of tapers = 20, yielding a frequency resolution 0f 0.5 Hz. Frequencies higher than 30 Hz were discarded and the resulting spectral power, excluding the 2% minimal and maximal outliers, was log scaled and color coded (black < blue < cyan < orange < red < dark red). If a NREM sleep episode was interrupted by 15 s or less (3 epochs), this interval was incorporated into the ongoing NREM sleep episode but omitting delta power computation. The resulting daily vector (12 values at 2-h time resolution) for NREM sleep delta power was normalized (*z*-scored: mean = 0, *SD* = 1) to compare the temporal profile among genotypes.

Mixed model ANOVA was chosen to evaluate between group factor “genotype” (two levels) and within group factors “light-dark schedule,” “phase,” “zeitgeber time” and “NREM sleep bin,” and factor interactions. Repeated-measures for within group comparisons were nested in “genotype”: “zeitgeber time” with 24 levels for sleep–wake state amounts, “light-dark schedule” with two levels (LD and SP), “phase” with two levels (rest and active), and “NREM sleep bin” that quantifies the normalized delta power of a given 15-s interval within the first 4 min of NREM sleep episodes numerated according to the time elapsed since the beginning of the episode (16 levels). Time series may also be collapsed at different temporal time resolution as described earlier (phase, 2-h blocks, hours and 15 s). *Post hoc* paired *t*-test (for within group) and unpaired *t*-test (for between group comparisons) were performed after significant ANOVA for the apropriate factor or factor interaction, significance level was set at 0.05.

Rayleigh’s test was used to estimate the significance of the daily mean angles of sleep–wake states. After obtaining group mean angle (Batschelet procedure), statistical significance was obtained by means of a second-order population analysis (Hotelling procedure) for each experimental condition. Second-order paired-sample parametric tests for mean angle comparisons were performed within genotypes (Zar, 1999). Watson-Williams mean angle tests for two samples was performed to compare between genotypes (Zar, 1999).

## Results

Representative records and corresponding state scoring obtained in WT and alpha9-nAchR KO mice are depicted in **Figure 1**. The automated scoring system is based on the generation of three distinctive clusters representing main sleep–wake states. The automated scoring procedure is further validated by visual scoring of 6 h. The supervised automated scoring procedure allowed us to identify the three main states and to generate the animal’s hypnogram with 5-s time resolution.

### Sleep–Wake State Quotas

State amounts of WT and alpha9-nAchR KO individuals obtained under LD and SP schedules are summarized in **Table 1**. Total amount of wakefulness, NREM sleep and REM sleep do not differ between alpha9-nAchR KO individuals and their WT littermates [mixed ANOVA, factor “genotype”: *F*_(1,14)_= 2.26, *p* = 0.129; *F*_(1,14)_= 3.39, *p* = 0.0870; and *F*_(1,14)_= 0.52, *p* = 0.481, for W, NREM sleep and REM sleep respectively]. Transference from a LD schedule to a SP do not significantly affect daily sleep quotas as estimated by the repeated factor “light-dark schedule” in the mixed ANOVA model [*F*_(1,14)_= 0.05, *p* = 0.82; *F*_(1,14)_= 0.00, *p* = 0.98; and *F*_(1,14)_= 0.25, *p* = 0.63 for W, NREM sleep and REM sleep respectively]. There are rest vs. active phase differences for each state among WT and KO individuals under LD and SP schedules [mixed ANOVA, repeated factor “phase”: *F*_(1,14)_= 58.8, *p* < 0.001; *F*_(1,14)_= 40.6, *p* < 0.001; and *F*_(1,14)_= 60.0, *p* < 0.001, for W, NREM sleep and REM sleep, without interaction with factors “genotype” and “light-dark schedule”]. Both WT and alpha9-nAchR KO individuals exhibit nocturnal phase preference of wakefulness and higher proportion of sleep states during the light phase under LD schedule. After transference to SP rest phase and active phase quotas of states remain almost unaffected.

**Table 1:** Daily, rest phase and active phase sleep–wake state quotas.

		Wild type		Alpha 9-nAChR KO	
		Mean	SEM	Mean	SEM
**Light-dark schedule**					
W	Daily	45.7	1.8	45.0	1.5
	Rest	39.9	2.3	37.3	1.5
	Active	51.5	2.5^∗^	52.7	2.8^∗^
NREM	Daily	46.3	2.1	48.2	1.4
	Rest	50.8	2.9	54.7	1.2
	Active	41.8	2.4^∗^	41.8	2.6^∗^
REM	Daily	8.0	0.7	6.8	0.7
	Rest	9.3	0.8	8.0	0.5
	Active	6.7	0.7^∗^	5.5	1.0^∗^
**Skeleton photoperiod**					
W	Daily	48.3	1.4	43.1	1.5
	Rest	43.2	1.6	37.8	1.2
	Active	53.3	2.2^∗^	48.3	2.2^∗^
NREM	Daily	44.6	1.7	49.8	1.7
	Rest	48.2	1.7	53.6	1.4
	Active	40.9	2.2^∗^	46.1	2.2^∗^
REM	Daily	7.1	0.5	7.1	0.7
	Rest	8.5	0.6	8.6	0.9
	Active	5.7	0.7^∗^	5.6	0.7^∗^

*Values correspond to the mean ± SEM obtained for 6 WT and 10 KO animals (% of corresponding recording time). ^∗^*P* < 0.05, paired *t*-test for active vs. rest phase comparisons.*

**FIGURE 2:**
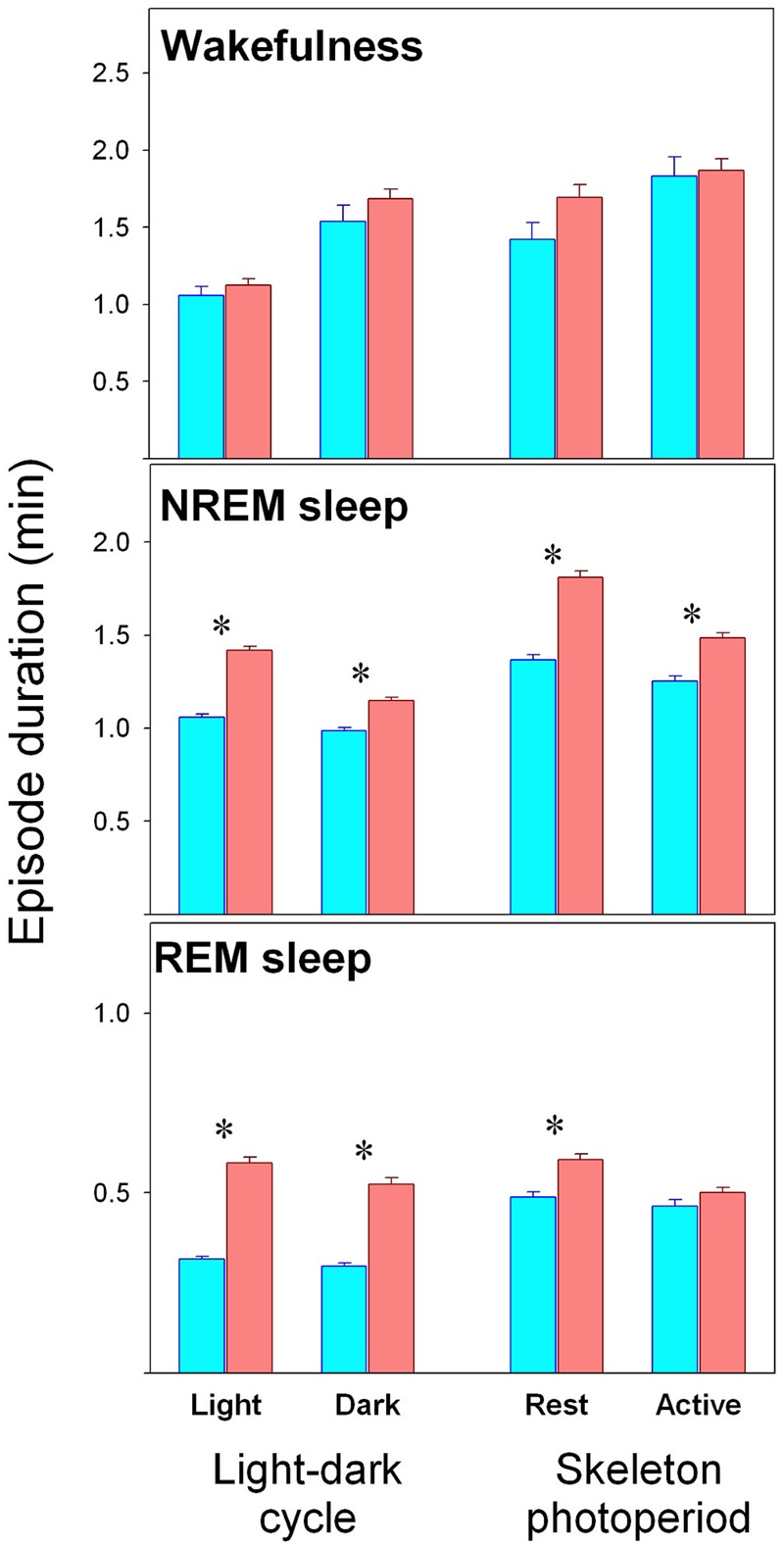
Duration of sleep–wake state episodes. Values correspond to mean (±SEM) duration of episodes obtained in WT (cyan bars) and KO (reddish bars) individuals as obtained during rest or active phases, under LD and SP as indicated in the abscissa. Significant WT vs. KO comparison (unpaired *t*-test, *p* < 0.05) is indicated by asterisks.

Sleep–wake state episode duration are presented in **Figure 2**. No differences are observed between KO and WT mice for wake episodes. On the contrary, longer NREM sleep episodes are observed among alpha9-nAchR KO subjects as compared to WT genotype during rest and active phases and under LD or SP schedules. Similarly alpha9-nAchR KO subjects exhibit longer REM sleep episodes under both rest and active phases under LD schedule and during rest phase under SP.

### Mean Curves and Circular Statistics of Sleep–Wake States

**FIGURE 3:**
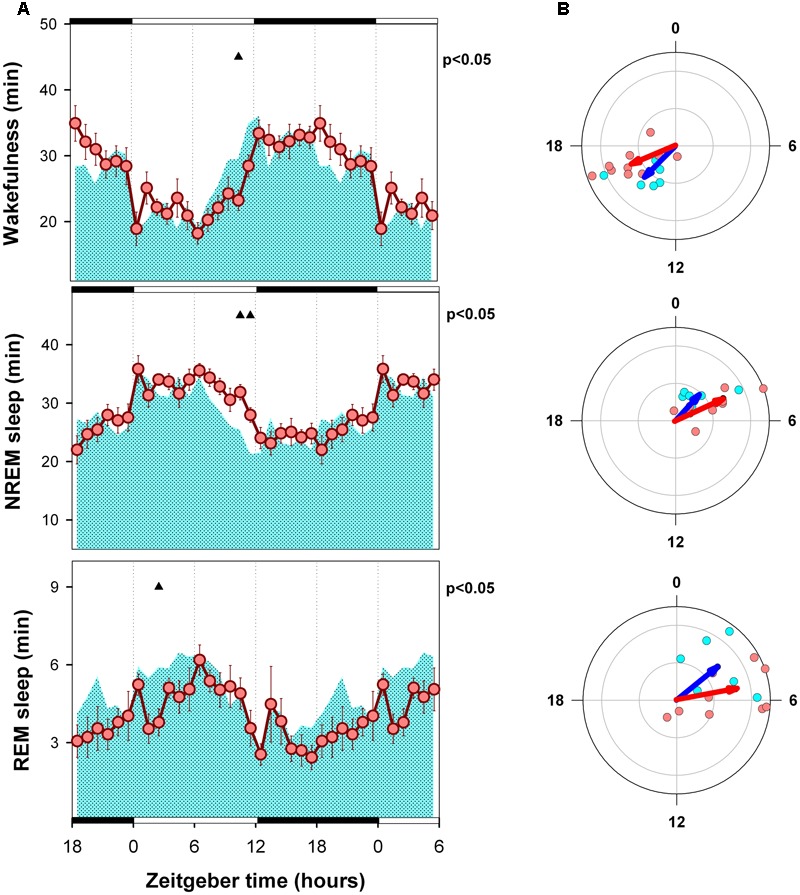
Mean curves of sleep–wake states of WT and KO mice under LD schedule conditions. **(A)** Cyan area (WT) and reddish circles (KO) correspond to the hourly mean time of the state (±SEM) obtained for 6 WT and 10 KO animals. Filled triangles under upper abscissa indicate significant WT vs. KO differences (two-tailed unpaired *t*-test) after significant mixed ANOVA for “Genotype” and “Zeitgeber time” factor interaction. LD schedule is depicted at the abscissa: white bars correspond to light phase under 12: 12 LD condition and 20-min light pulses under SP, black bars correspond to darkness intervals. **(B)** Blue and red arrows correspond to second order mean vectors (Hotelling analysis of angles) obtained for W, NREM sleep and REM sleep in WT and KO animals. Cyan (WT) and reddish (KO) dots correspond to averaged individual acrophases for the corresponding vector. Radial ordinate corresponds to the magnitude (r) of the mean vector (total radius = 0.3).

**FIGURE 4:**
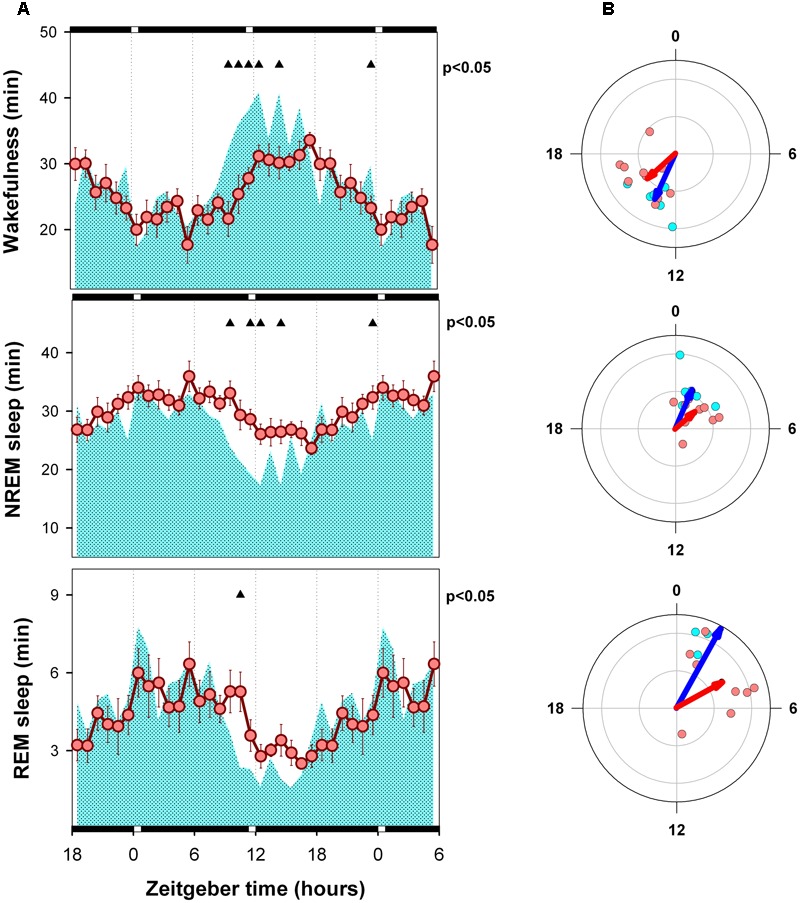
Mean curves of sleep–wake states of WT and KO mice under skeleton photoperiod. **(A)** Cyan area (WT) and reddish circles (KO) correspond to the hourly mean time of the state obtained for 6 WT and 10 KO animals. Filled triangles under upper abscissa indicate significant WT vs. KO differences (two-tailed unpaired t-test) after significant mixed ANOVA for “Genotype” and “Zeitgeber time” factor interaction. **(B)** Blue and red arrows correspond to second order mean vectors (Hotelling analysis of angles) obtained for W, NREM sleep and REM sleep in WT and KO animals. Cyan (WT) and reddish (KO) dots correspond to averaged individual acrophases for the corresponding vector. Radial ordinate corresponds to the magnitude (r) of the mean vector (total radius = 0.3). Illumination schedule is indicated on abscissa: white bars correspond to 20-min light pulses under SP, black bars correspond to darkness intervals.

Hourly means of each state are presented in **Figures 3A, 4A**, for LD and SP conditions respectively. Wakefulness of WT and alpha9-nAchR KO exhibit similar temporal profiles with minimal differences under LD schedule [mixed model ANOVA “zeitgeber time”^∗^ “genotype” interaction, *F*_(23,322)_= 2.01, *p* = 0.015]. Compared to LD condition, WT individuals exhibit incremented amounts of wakefulness under SP during an interval close to the evening light pulse (4.8 and 5.8 min increment at ZT 12 and ZT 13, paired *t*-test *p* = 0.043 and *p* = 0.037 respectively). Similarly, compared to alpha9-nAchR KO, WT mice present higher values of wakefulness in the ZT 9 to ZT 14 interval under SP as depicted in **Figure 4A**. “Genotype” and “zeitgeber time” interactions are also present for NREM sleep and REM sleep mixed model ANOVAs [*F*_(23,322)_= 1.83, *p* = 0.032; and *F*_(23,322)_= 1.72, *p* = 0.036 respectively]. As observed for wakefulness, NREM and REM sleep differences between WT and KO mice under LD condition are minimal. Under SP sleep state differences between WT and KO mirror evening increments in wakefulness with corresponding NREM sleep reductions.

Acrophase vectors mean angles of sleep–wake states were estimated by means of Batschelet procedure (**Figures 3B, 4B**). Second order Hotelling tests was significant (*p* < 0.05) for each state under each light-dark schedule and genotype. Under LD schedule wakefulness acrophase of WT and KO animals were located at ZT 14.9 and 16.5 (Watson-Williams comparison for two samples, *p* > 0.05). Under the same condition NREM sleep acrophases of WT and KO subjects were located at ZT 2.8 and ZT 4.3 (Watson-Williams comparison for two samples, *p* > 0.05) and that of REM sleep were ZT 3.4 and ZT 5.3 (Watson-Williams comparison for two samples, *p* > 0.05). After transferred to SP no significant differences were observed in wake, NREM sleep and REM sleep acrophases respect to LD schedule (within genotype, paired sample, comparisons for mean angles *p* > 0.05) among WT and KO animals. Furthermore, Watson-Williams test demonstrate no differences for sleep–wake state acrophases between WT and KO under SP.

**FIGURE 5:**
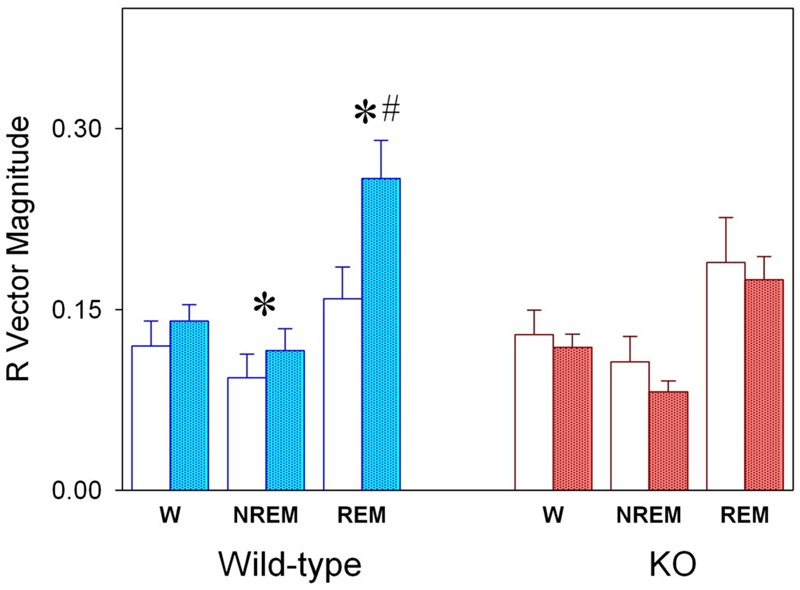
Magnitude (R) of mean vector for the sleep–wake cycle states of WT and KO genotypes under LD and SP schedules. Values correspond to averages *R* values (±SEM) obtained for each state for WT (blue columns, *n* = 6) and KO (red columns, *n* = 10) under LD (open columns) and SP (filled columns) schedules. ^∗^*P* < 0.05, two-tailed paired *t*-test for LD vs. SP schedule; ^#^*P* < 0.05, two-tailed unpaired *t*-test for WT vs. KO groups.

Sleep state vector magnitude (R) of WT and KO animals under LD and SP are compared in **Figure 5**. Whereas alpha9-nAchR KO exhibit no significant changes after transferred from LD to SP condition, WT animals exhibit significant increments in R vector magnitude for NREM (paired *t*-test, *p* = 0.045) and REM sleep (paired *t*-test *p* = 0.045). The R vector magnitude estimated for WT REM sleep is higher than that of KO group (unpaired *t*-test, *p* = 0.029).

#### Delta NREM Sleep EEG Activity

**FIGURE 6:**
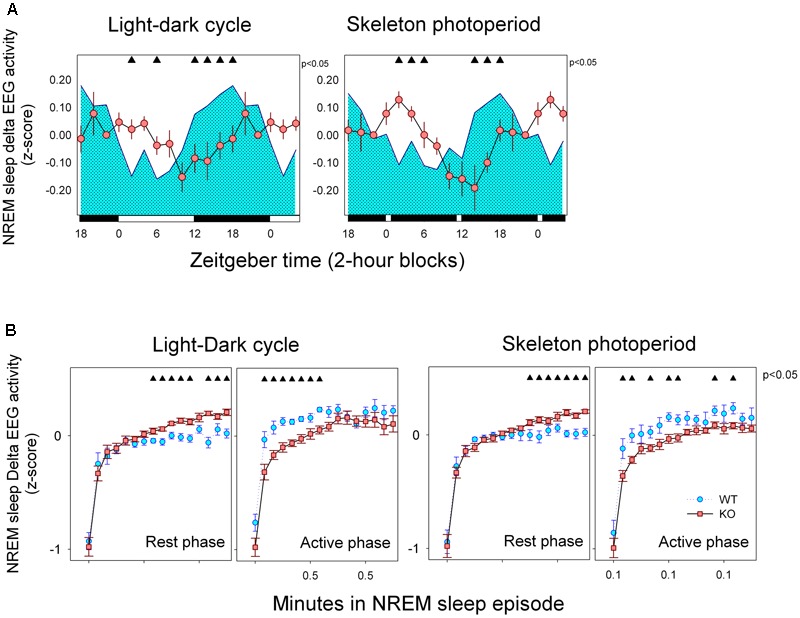
**(A)** Mean curves of NREM sleep delta activity. Cyan area (WT) and reddish circles (KO) correspond to the 2-h mean normalized delta EEG activity in NREM sleep (±SEM) obtained for 6 WT and 10 KO animals. Filled triangles under upper abscissa indicate significant WT vs. KO differences (two-tailed unpaired *t*-test) after significant mixed ANOVA for “Genotype” and “Zeitgeber time” factor interaction. LD schedule is depicted at the abscissa. **(B)** Build-up of delta EEG activity within NREM sleep episodes. Data correspond to mean (±SEM) of normalized delta activity of WT (*n* = 6, cyan symbols) and KO (*n* = 10, reddish symbols) animals under LD and SP as indicated. Data are presented with 15-s time resolution. Data were analyzed within 12-h blocks corresponding to rest and active phases. Filled triangles indicate differences between genotypes (unpaired *t*-test comparisons, *P* < 0.05, after mixed ANOVA for factors epoch and genotype within each block).

Normalized (daily based *z*-score) values of delta EEG activity was estimated for 2-h blocks (**Figure 6A**). Mean curves of normalized NREM sleep EEG delta power density were obtained for WT and KO animals under LD and SP conditions. Only consolidated (>2-min) NREM sleep episodes were included for statistical analysis. Mean curves of KO and WT group profiles were compared under LD and SP schedules by means of unpaired *t*-test after significant mixed ANOVA for “zeitgeber time” ^∗^ “genotype ” interaction [*F*_(11,143)_ = 6.91, *p* < 0.001]. No significant effect of factor “light-dark schedule” nor “light-dark schedule” ^∗^ “genotype” or “light-dark schedule” ^∗^ “zeitgeber time” interactions were obtained. Normalized NREM sleep EEG delta power densities exhibit different time profiles when comparing WT and alpha9-nAchR KO animals. Higher densities of delta NREM sleep EEG was observed during rest phases for KO animals whereas WT individuals exhibit maximal values during the active phase under both LD and SP schedules.

To further analyze the dynamics of NREM sleep EEG delta power density, we explored the build-up of NREM sleep delta activity within consolidated (>2 min) NREM sleep episodes under LD and SP during rest and active phases (**Figure 6B**). A mixed ANOVA with three repeated measures (“light-dark schedule,” “phase” and “NREM sleep bin”) nested in genotype, was performed. Factor “NREM sleep bin” was highly significant [*F*_(15,195)_ = 128.85, *p* < 0.001) and a significant interactions were obtained for “genotype” ^∗^ “phase” [*F*_(1,13)_ = 15.21, *p* = 0.002]. Typically, delta activity increases during a brief initial rising interval after state transition up to a plateau that starts after 1.5 min among consolidated NREM sleep episodes. Normalized delta activity during the plateau was consistently higher for alpha9-nAchR KO animals respect to WT individuals during rest phase under both LD and SP conditions (**Figure 6B**). On the other hand, WT individuals exhibit a rapid increase in delta EEG activity during NREM sleep episodes that occur during active phase under both light-dark schedules. Under SP WT animals sustain higher values of delta activity during the plateau region (after 1.5 min of sustained NREM sleep).

## Discussion

### Sleep–Wake State Quotas and Phase Preference

Wild type and alpha9-nAchR KO mice exhibit similar amounts of sleep–wake states that are almost unaffected by light-dark schedule as observed after the transference from LD to SP. State quotas do not depart from values reported in diverse mice strains obtained under LD conditions and constant darkness (Deboer et al., 2007). The studied strain exhibit nocturnal phase preference of wakefulness as correspond to the typical mouse chronotype, as under LD 56.4 and 58.7% of wakefulness is concentrated during the active phase in WT and alpha9-nAchR KO mice respectively. The transference from LD to SP do not significantly affect day/night wakefulness distribution of WT and KO (**Table 1**).

Sleep (NREM and REM) states are concentrated during the light phase under LD schedule and the diurnal preference persisted after the transference to SP. To avoid transient and unspecific effects of the light-dark schedule transference, records under SP were obtained after at least 3 days of the new light-dark regime. Diurnal phase preference of sleep states is a common characteristic of mice strains studied under LD conditions (Franken et al., 1999). Experiments performed under free running conditions (constant darkness) obtained in mice, have largely demonstrated that sleep states concentrate during the subjective (or circadian) day as correspond to nocturnal-active chronotype (Deboer et al., 2007).

The stability of daily NREM and REM sleep quotas is assumed to be dependent on sleep homeostatic processes that compensate for losses and gains of the affected state (Deboer, 2013). The almost equal amount of daily sleep and wake states observe among WT and KO littermates and its stability across different light-dark schedules suggest that alpha9-nAchR is not relevant for sleep homeostatic processes.

### Circadian Modulation of Sleep–Wake State

To evaluate circadian modulation of the SWC we opted to perform a two-pulse symmetric skeleton photoperiod protocol. The rationale after the skeleton photoperiod design is to unmask the endogenous phase preference modulated by the circadian process by maintaining the SWC within the 24-h frame determined by the light entrainable oscillator and simultaneously eliminating the direct effect of the light on sleep–wake state expression. The photic entrainment is maintained by morning and evening light pulses timed at corresponding local time of lights on and off under 12:12 LD cycle. One evident limitation of SP protocols regarding circadian studies is that it is not possible to estimate the endogenous period of the circadian rhythm as occurs in free-running experiments under constant condition. In this report we sacrificed the evaluation of the endogenous period because of the much longer experimental time required to confidently estimate circadian time (at least 1 week per animal), and opted to compare the acrophase stability across photoperiod conditions.

Acrophases of sleep wake states are consistent with the expected nocturnal active chronotype of mice. Wake acrophases of WT and KO animals are centered close to midnight (ZT 14.9 and 16.5 respectively) and that of sleep states are located in the first quadrant of the LD cycle (ZT 0 to ZT 6) under LD (**Figure 3B**). On the other hand, the strength of circadian modulation estimated by R vector appears to be similar among genotypes under LD schedule (**Figure 4**). Consistently, the mean curves of each state obtained under LD schedule exhibit minimal differences (**Figure 3A**).

After transference to SP conditions the acrophase of sleep–wake states exhibit a minor and no significant phase advance (1–1.5 h) among WT and KO animals (**Figure 4B**). By examining state mean curves it result evident that compared to KO animals, WT mice exhibit higher amounts of wakefulness in a 6-h interval centered at the evening pulse of light (**Figure 4A**). The mean curve of NREM sleep of WT mice mirrors wakefulness increments by presenting lower amounts than that of alpha9-nAchR KO animals at ZT 9, 10, 11, and 14. Minor differences (at ZT 9) are observed for REM sleep between KO and WT by comparing hourly mean curves. Interestingly, the amplitude of NREM sleep and REM sleep mean curves (R vector) is significantly incremented under SP among WT animals respect to LD conditions as depicted in **Figure 5**, and compared to KO mice, WT REM sleep R vector exhibit stronger circadian modulation.

Mohammadi et al. (2017) reported a crepuscular bimodal pattern of activity among WT individuals, measured by the “corner visit method” that is absent among KO mice, characterized by a monophasic nocturnal profile, suggesting an altered circadian modulation of activity. Our results on the temporal organization of wakefulness, that with caution could be assimilated to activity, do not coincide with the described crepuscular pattern as both WT and KO animals exhibit a unimodal profile of wakefulness under LD and SP conditions. Discrepancies between both studies may be related to different recording strategies or housing conditions (light intensity, isolation, etc.).

### Photic Masking

The fact that the acrophases of sleep–wake states were unaffected by LD to SP transference (**Figures 3, 4**) supports that the circadian timing is not affected by factor genotype. Nevertheless, transient differences between light-dark schedule and genotype are evident from the analysis of state mean curves. In the pigmented rat, light to dark transitions trigger wakefulness increments at any time of the 24-h period (Benca et al., 1998). In mice, light stimulation suppresses general activity and dark pulses favor transient state transitions in C57/BL6 mice (Deboer et al., 2007). Consistently, the transient increment of wakefulness observed among our WT animals at late rest phase may be interpreted as the unmasking of a wake promoting circadian signal at dusk, that correspond to the beginning of the active phase. Alpha9-nAchR KO mice do not exhibit the positive masking of wakefulness at dusk. Whereas WT mice exhibit an increment of 31 min of wakefulness in the ZT9-ZT14 interval (paired *t*-test, *p* = 0.054), KO animals present a no significant decrement of 6.1 min (*p* > 0.1).

It is well known that illumination conditions may directly affect the stability and dynamics of sleep–wake states among rodents by means of photic masking processes, that depends on the integrity of retinofugal projections provided by intrinsically photosensitive retinal ganglion cells upon sleep and wake state generator centers distributed in the hypothalamus, basal forebrain and brainstem (Lupi et al., 2008; Pickard and Sollars, 2011; Hubbard et al., 2013). Recent evidences suggest two independent photic masking processes, one arousal-promoting process that depends on M1 melanopsin containing ipRGCs, and a sleep-promoting process that depends on non-M1 melanopsin containing ipRGCs (Pilorz et al., 2016). M1 ipRGCs favors arousal in response to blue light stimulation (wave length centered at 470 nm) and also participate in photic entrainment by directly projecting to the hypothalamic suprachiasmatic nucleus. Our data do not support the involvement of alpha9-nAchR on M1 operation (positive masking of wakefulness and entrainment) because of the observed acrophase robustness under LD and SP among WT and KO animals (**Figures 3, 4**), and because the increment of wakefulness among WT individuals at late rest phase occurred under SP, in the absence of light. On the other hand, the concurrent increase of wakefulness and NREM sleep decrease at late rest phase among WT animals could be explained by the lack of positive masking of sleep under SP. In other words, wakefulness is suppressed among WT animals at late rest phase under LD schedule by the positive masking of sleep exerted by the presence of light, a process that appears to be absent among KO littermates. Evidences support that positive masking of sleep occurs in response to green light (wave length centered at 530 nm). The cellular substrate that sustain positive sleep photic masking may include green cones, rods, and non-M1 ipRGCs (Pilorz et al., 2016). The recently described presence of alpha 9-nAchR within bipolar, amacrine and retinal ganglion cells (Smith et al., 2014) may be related to the impairment of photic masking among KO animals. In sum, we propose that the observed increment in wakefulness at dusk among WT individuals reports the unmasking of an endogenous (circadian driven) arousal process that under a continuous LD schedule is masked by a alpha 9-nAchR-dependent sleep promoting process.

### Delta NREM Sleep EEG Activity Modulation and Microstructure of Sleep

In sharp contrast to humans, whose SWC exhibit a biphasic organization consisting in one long wake period followed by one consolidated sleep episode, that of small mammals is typically polyphasic, with repetitive sequences of short episodes of wake-NREM sleep-REM sleep (sleep cycles) occurring during the whole day (Le Bon et al., 2007). Compared to KO animals, WT exhibit an overall shorter duration of NREM sleep and REM sleep bouts under both LD and SP schedules. Although sleep episode consolidation has been associated to homeostatic processes that stabilize sleep bouts (Deboer, 2013), no evidences of sleep debt can be extracted from our data as sleep quotas of WT and KO animals demonstrated to be similar as noted above. Sleep deprivation experiments should be performed to evaluate the sleep homeostatic competence of the studied mice strains. Alternatively, it has been reported that environmental challenges such as low ambient temperature may determine high sleep fragmentation as a physiologic response to a live threatening condition (Cerri et al., 2005). Prolonged sleep episodes observed among KO respect to WT individuals may be related to the impairment of multimodal (somatosensory, sensory and metabolic) stimuli that normally interrupt sleep bouts. If this is true, alpha 9-nAchR-dependent signaling processes may be of relevance to monitor potentially harmful environmental variables during sleep.

One notable finding in our study is the difference in the temporal profile of normalized delta NREM sleep EEG activity between WT and alpha 9-nAchR KO littermates (**Figure 6A**). Delta NREM sleep activity of KO animals exhibit higher values during rest phases under both LD and SP schedules in contrast to WT mice whose higher values occurred in the active phase. In addition to the discordant temporal profile of NREM sleep delta activity, alpha9-nAchR KO animals exhibit higher consolidation of sleep episodes at rest and active phases, phenomenon particularly evident for NREM sleep, as compared to the fragmented sleep episodes of WT littermates (**Figure 2**).

Because most of NREM sleep EEG delta power temporal course in undisturbed sleep records and after sleep deprivation is dependent on the cumulated amount of wakefulness NREM sleep delta power is used as an index of homeostatic sleep need (Deboer, 2013). In rats and among most mice strains, there is a delta activity increment during the active phase as a function of wake time accumulation that is followed by the typical decay of NREM sleep delta during rest phase that parallels the dissipation of sleep need (Franken et al., 1999; Huber et al., 2000). Among our experimental animals only the alpha9-nAchR KO group appear to follow the expected profile of delta activity according to the sleep homeostatic process, nevertheless the detailed inspection of the buildup of delta activity (**Figure 6B**) within the single NREM sleep episode can help us to understand the KO vs. WT discrepancy.

It is well known that delta EEG activity of NREM sleep is affected by sleep fragmentation. Cortical recruitment into slow-wave sleep starts as a gradual process after transition to NREM sleep. Typically, the buildup of delta activity within NREM sleep episodes follows an initial rising interval that lasts 1–3 min followed by a plateau where maximal cortical synchronization could be sustained (Trachsel et al., 1988), that in our experimental animals start after 2 min (**Figure 6B**). High fragmentation of NREM sleep episodes interferes with the full expression of delta waves as the episode is frequently interrupted before the delta plateau is reached. There is a higher fragmentation of NREM sleep episodes among WT (mean duration of episodes was 1.1 min) respect to KO mice (mean duration 1.5 min) and only 13% of NREM sleep episodes of WT mice are longer than 2 min as compared to 20% observed among KO animals. It could also be seen in **Figure 6B** that the upper delta plateau is higher for KO animals during rest phase under LD and SP schedules. Longer duration and higher delta plateaus during the rest phase are characteristic of NREM sleep episodes of alpha9-nAchR KO animals, a fact that may explain the diurnal phase preference of NREM sleep delta activity of the genotype. On the other hand, a faster buildup of delta EEG activity can be seen among WT individuals during the nocturnal (or active) phase, so that plateau levels of delta activity may be approached before 2 min of duration of NREM sleep. NREM sleep episodes lasting 1 to 2 min correspond to 26.5 and 25.9% among WT and KO mice respectively. In sum: as WT animals may efficiently predate on shorter NREM sleep episodes to develop delta EEG during active phase as compared to KO littermates, higher levels of normalized delta NREM sleep EEG activity are obtained during the active phase compensating the relatively lower levels of deep NREM sleep expressed during the rest phase.

## Conclusion

Circadian and homeostatic processes appears to be independent of alpha 9-nAChR expression in mice as total sleep–wake state quotas, phase preference and circular distribution of sleep–wake states are unaffected among KO mice. Retinal alpha 9-nAChR may play a relevant role in the positive masking of sleep by light.

## Author Contributions

AO-G, NM-L, and PD designed research; NM-L and JE performed research; AB, JD, and PD contributed with designing analytic tools ; NM-L, JE, JD, and AB analyzed data; AO-G, JE, JD, NM-L, and AB interpreted data; NM-L, JE, JD, AB, PD, and AO-G revised critically the manuscript; NM-L, AO-G, JD, AB, and PD identified important intellectual content; AO-G wrote the paper; PD, NM-L, AB, JD, and JE approved the final version and evaluated the accuracy and integrity of the work.

## Conflict of Interest Statement

The authors declare that the research was conducted in the absence of any commercial or financial relationships that could be construed as a potential conflict of interest.
